# Analgesia during Labor and Vaginal Birth among Women with Severe Maternal Morbidity: Secondary Analysis from the WHO Multicountry Survey on Maternal and Newborn Health

**DOI:** 10.1155/2019/7596165

**Published:** 2019-02-13

**Authors:** Marcio A. Souza, Jose P. S. Guida, Jose G. Cecatti, João P. Souza, Ahmet M. Gulmezoglu, Ana P. Betran, Maria R. Torloni, Joshua P. Vogel, Maria L. Costa

**Affiliations:** ^1^Department of Obstetrics and Gynecology, School of Medical Sciences, University of Campinas, Campinas, Brazil; ^2^Department of Social Medicine, Ribeirao Preto Medical School, University of Sao Paulo, Ribeirao Preto, Sao Paulo, Brazil; ^3^The UNDP/UNFPA/UNICEF/WHO/World Bank Special Programme of Research, Development and Research Training in Human Reproduction, Department of Reproductive Health and Research, World Health Organization, Geneva, Switzerland; ^4^Federal University of Sao Paulo, Sao Paulo, Brazil; ^5^Maternal and Child Health Program, Burnet Institute, Melbourne, Australia

## Abstract

**Aim:**

To evaluate the use of analgesia for vaginal birth, in women with and without severe maternal morbidity (SMM) and to describe sociodemographic, clinical, and obstetric characteristics and maternal and perinatal outcomes associated with labor analgesia.

**Methods:**

Secondary analysis of the WHO Multicountry Survey on Maternal and Newborn Health (WHO-MCS), a global cross-sectional study performed between May 2010 and December 2011 in 29 countries. Women who delivered vaginally and had an SMM were included in this analysis and were then divided into two groups: those who received and those who did not receive analgesia for labor/delivery. We further compared maternal characteristics and maternal and perinatal outcomes between these two groups.

**Results:**

From 314,623 women originally included in WHO-MCS, 9,788 developed SMM and delivered vaginally, 601 (6.1%) with analgesia and 9,187 (93.9%) without analgesia. Women with SMM were more likely to receive analgesia than those who did not experience SMM. Global distribution of SMM was similar; however, the use of analgesia was less prevalent in Africa. Higher maternal education, previous cesarean section, and nulliparity were factors associated with analgesia use. Analgesia was not an independent factor associated with an increase of severe maternal outcome (Maternal Near Miss + Maternal Death).

**Conclusions:**

The overall use of analgesia for vaginal delivery is low but women with SMM are more likely to receive analgesia during labor. Social conditions are closely linked with the likelihood of having analgesia during delivery and such a procedure is not associated with increased adverse maternal outcomes. Expanding the availability of analgesia in different levels of care should be a concern worldwide.

## 1. Introduction

Many physical, psychological, and cultural factors may shape women's individual experience during childbirth [1, 2]. Physical pain experience during labor can be very intense for many women and it is one of the factors underpinning women's preferences for caesarean section worldwide [3–5].

Pharmacological analgesia is different medical interventions drug-based to relief physical pain during childbirth. They can be systemic, when opioids are provided parenterally, or regional, when a neuraxial blockage is performed through epidural or spinal injection of opioids and anesthetic. Also, local anesthesia can be used to block pudendal nerve and other perineum tissues. Use of inhaled agents as nitrous oxide has the benefit to preserve maternal mobility; however its effectiveness is lower than neuraxial blockage. Anesthetic choice is mainly determined by women's clinical conditions, anesthetist skills, and facility condition to offer any of those previous options [6].

According to the American College of Obstetricians and Gynecologists, pharmacological analgesia is a safe intervention to relieve pain and physical discomfort and it is unacceptable that this intervention is not provided to a woman who requests it, unless a medical contraindication to the procedure exists [6, 7], or even due to a lack of personnel or infrastructure in low resourced environments. The availability of such intervention shows great variations among different settings [8, 9], and there are initiatives to qualify nurses to participate in the labor pain relief management in order to expand its availability [6].

Pharmacological analgesia provided during delivery is associated with higher satisfaction rates during labor and it also increases the desire for induction of labor in subsequent pregnancies [10]. However, with the current trends of changes in population characteristics, such as advanced maternal age at first pregnancy, obesity, and complexity of other medical conditions that disturb pregnancy, offering pharmacological analgesia for labor and delivery may be challenging [11].

Women with such characteristics are at increased risk of maternal morbidity and adverse perinatal outcomes. Severe maternal morbidity (SMM) affects millions of women every year all over the world during pregnancy, childbirth, or the postpartum period [12]. The continuum of SMM severity has been established by the World Health Organization (WHO) [13] and its criteria have been validated [14]. SMM comprises Potentially Life-Threatening Conditions (PLTC), Maternal Near Miss (MNM), and Maternal Death (MD) [13, 15]. The study of maternal morbidity and factors associated with worse outcomes can enable future interventions towards the improvement of maternal health and the identification of such conditions is key to ascertain adequate and timely care during childbirth.

The use of analgesia in women with SMM has not been explored previously. SMM cases have increased rates of cesarean sections [14]; however, when induction is possible or if there is spontaneous labor, pharmacological analgesia can be an effective intervention, to enable a better experience for the woman and a better clinical control of vital signs.

PLTC such haemorrhagic or hypertensive disorders, when not timely diagnosed or adequately treated, deteriorate to MNM or even MD [13]. Women with pharmacological analgesia are in more skilled facilities and are also more watched by medical team, allowing an early diagnosis of those conditions and providing fast medical care, preventing them to evolve to a worse outcome.

The aim of this study is to explore the use of analgesia among women with and without SMM and assess maternal and perinatal outcomes among women with SMM that delivered vaginally, comparing results between those who had and those who did not have analgesia during labor.

## 2. Methods

This is a secondary analysis of the World Health Organization (WHO) Multicountry Survey on Maternal and Newborn Health (WHO-MCS), whose methodological details have been previously published [16, 17]. Briefly, it was a global cross-sectional study performed between May 1^st^, 2010, until Dec 31^st^, 2011, and included 359 facilities in 29 countries in Latin America, Africa, Asia, and Middle East. Data were obtained from medical records by trained researchers and it was stored in a web-based data management system. For the WHO-MCS, all women who delivered in the selected centers during data collection period were considered, and also women who had severe maternal morbidity or maternal death up to seven days after delivery or abortion were included and had their medical records reviewed. Data of fetal and newborn's conditions were also retrieved. The protocol of the survey was approved by the Research Project Review Panel at the UNDP/UNFPA/UNICEF/WHO/World Bank Special Programme of Research, Development and Research Training in Human Reproduction and by the WHO Ethical Review Committee as well as the relevant ethical clearance bodies in participating countries and facilities [16].

In the original study, a total of 314,623 women were included, with a prevalence of 7.3% (23,015) of PLTC, 0.81% (2,538) of MNM, and 0.15% (486) of MD reported, with a rate of Severe Maternal Outcome (SMO) of around 1% [16].

For the current analysis, only women who delivered vaginally were selected from the WHO-MCS. Women who had a cesarean section, abortion, or ectopic pregnancies, deliveries before arrival at the health facility, macerated stillbirths, gestational age below 22 weeks, or fetal birthweight of less than 500 grams were excluded.

Among those who delivered vaginally, we further selected women who had SMM. To assess maternal morbidity, we applied the WHO criteria for SMM [13], classifying each case according to its severity as PTLC and MNM and related with clinical conditions of specific diseases, intervention-based criteria or due to organ system dysfunction. Women with the most severe conditions, MNM and MD, are grouped as Severe Maternal Outcome (SMO).

**Figure 1:**
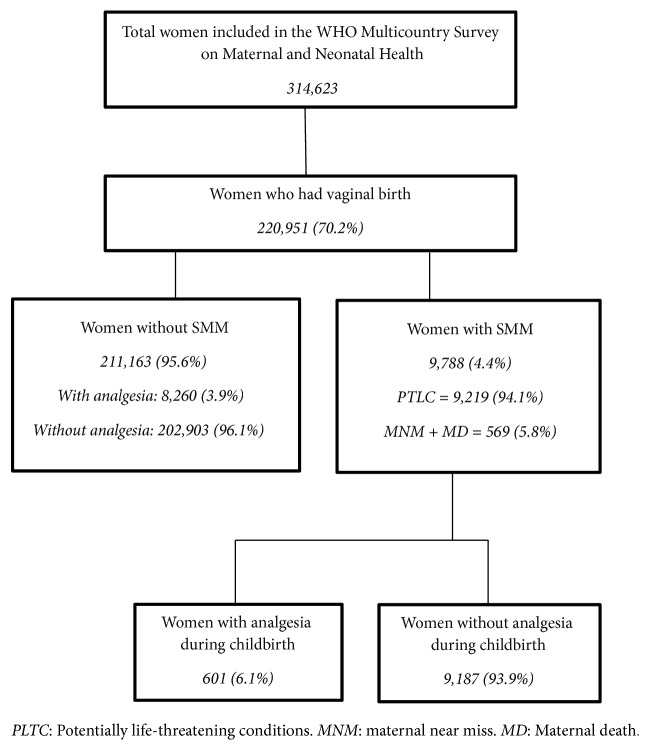
Flowchart showing the inclusion of women in this analysis.

To assess the impact of analgesia for pain relief during childbirth, we divided women who delivered vaginally and experienced SMM into two groups, according to the use of analgesia. We considered any kind of analgesia (systemic, epidural, or spinal). However, the study did not capture other nonpharmacological forms of pain relief during childbirth as water immersion or massage therapy. A flowchart of women included in this analysis is provided in Figure 1. We first evaluated the prevalence of SMM in different geographic regions of the world, among vaginal deliveries. Countries included in this analysis were divided into three groups, according to geographic distribution in continents: Latin America, Asia, and Africa. Then we compared the prevalence of analgesia among SMM cases by region.

We further assessed data on sociodemographic and obstetric characteristics, such as age, marital status, education, parity, the occurrence of a previous cesarean delivery, and pre-existing clinical conditions. We also evaluated data of perinatal outcomes: time and type of delivery, fetal presentation, occurrence of postpartum hemorrhage, newborn wellbeing at delivery, weight, neonatal complication, and admission to intensive care unit (ICU). In addition, we assessed data on Neonatal Near Miss (birthweight < 1750g and/or 5th min Apgar <7 and/or GA< 33 weeks) and neonatal death. We further categorized the MNM criteria according to the specific organic system dysfunction present and we assessed the criteria for potentially life-threatening conditions during delivery or childbirth.

Data were processed and analyzed using SPSS 16.0 software. Prevalence of the conditions in both groups was obtained and they were compared using the prevalence ratio and its 95% confidence interval. A multivariate analysis using Poisson regression to assess factors independently associated with SMO among women with SMM was also performed. To define which variables would be considered in the multivariate analysis, we consulted members of the research team who are also clinical obstetricians, since we could not find any previous literature approaching analgesia and maternal morbidity. In the model considered, maternal age, marital status, education, number of previous births, number of previous cesarean-sections, previous maternal comorbidities, preterm delivery, multiple pregnancy, fetal presentation, low birth weight, congenital malformation, and analgesia were considered as predictor variables. Results were presented in accordance with the STROBE statement [18].

## 3. Results

A total of 314,623 women were included in the WHO-MCS, and 220,951 (70.2%) of them had a vaginal birth. Of those, 9,788 (4.4%) were diagnosed with SMM and were therefore included in this analysis. Among those women, 601 (6.1%) had analgesia for pain relief during childbirth and 9,187 (93.9%) did not receive analgesia; among those without SMM, only 3.9% had analgesia (p-value < 0.01). Figure 1 shows the flowchart for inclusion in the current analysis and also presents the occurrence of PTLC and MNM+MD.

SMM rates were broadly similar in the three regions, with a prevalence ranging from 4.2% in Asia and Africa and reaching 5.5% in Latin America (Table 1). Data on the use of analgesia in women with SMM is shown in Table 2. Africa was the region with less analgesia among SMM cases delivering vaginally (0.3%). Among the three regions included in the study, Latin America used more analgesia for pain relief among SMM cases with 12.5% of women receiving this procedure.

We further compared sociodemographic, obstetric, and clinical characteristics within SMM cases with and without analgesia, in order to understand the factors associated with this intervention (Table 3). Majority of women in the analysis were 20–34 years old and married, and age distribution between the two groups was similar. Neither age nor marital status was associated with the use of analgesia. Higher education was associated with increased analgesia among women with SMM, and women who had 12 or more years of education were over 7 times more likely to receive analgesia. Multiparous women were significantly less likely to receive analgesia, while cases with a history of previous cesarean section had a higher probability of analgesia for vaginal birth.

MNM or MD are classified as Severe Maternal Outcome (SMO). Among women with SMO, comparing cases with and without analgesia, most organ systems presented a similar prevalence of dysfunction. Coagulation and uterine-related dysfunctions were significantly more prevalent in the analgesia group, and women with those dysfunctions were 2.15 and 5.66 times more likely to receive analgesia, respectively. There was no significant difference in the prevalence of MD between cases with and without analgesia. Data is shown in Table 4.

When considering the criteria for PLTC, among women who received analgesia, 233 (38.8%) had postpartum hemorrhage, and this frequency was similar in the other group (26.9%). Another frequent condition was preeclampsia that affected 145 (24.1%) women who received analgesia and 2,385 (26.0%) of those who did not receive it. Women with heart or lung diseases were significantly more likely to receive analgesia (prevalence ratio of 1.88 and 1.74, respectively), while women with HIV or eclampsia were less likely to have analgesia for vaginal birth (prevalence ratio of 0.08 and 0.32, respectively) (Table 5).

Perinatal outcomes in the studied population were similar in both groups for a low 5-minute Apgar score, neonatal complications, admission to neonatal ICU, and neonatal death until the 7^th^ day after birth, the occurrence of any adverse perinatal outcome and neonatal near miss. However, fresh stillbirths and perinatal deaths were significantly lower among women who received analgesia (prevalence ratio of 0.37 and 0.45, respectively), as well as small for gestational age babies (prevalence ratio of 0.64). The conditions preterm birth, multiple pregnancies, fetal presentation, and low birth weight were not associated with the use of analgesia while delivering a baby with a congenital malformation was associated with the use of analgesia (Table 6).

In the multivariate analysis, we aimed to assess which factors were independently associated with SMO (MNM and MD), among all those who had SMM. Our model tested 12 variables, as previously explained. Of those, education (more than 8 years) was a protective factor (prevalence ratio of 0.43) for SMO, while multiple pregnancies, preterm birth, low birthweight, and multiparity were associated with a higher occurrence of SMO. Data is shown in Table 7.

## 4. Discussion

Our analysis assessed the use of analgesia for pain relief during vaginal birth among women who experienced SMM, associated factors, and outcomes using a large WHO database (Multicountry Survey on Maternal and Newborn Health). To the best of our knowledge, this is the first study to specifically address analgesia among women with SMM.

SMM occurrence was similar in the different world regions evaluated; however, the use of analgesia in Africa was less prevalent than in Asia and Latin America. Women with higher education level, with a previous cesarean section and with fetus with congenital anomalies were more likely to give birth with analgesia, while multiparous women were less like to receive it. Coagulation and uterine-related diseases were the main causes of MNM and also more associated with analgesia use. Among women who experienced PTLC, those who had HIV and eclampsia were less likely to receive analgesia, while women with cardiac or lung disease were more likely to receive it.

SMM is a relatively new concept that has been widespread due to the efforts of the WHO to assess this phenomenon in different settings and countries [19, 20]. A list of PTLC and MNM criteria can be assessed by any obstetrician to classify a case under its care as a SMM case to receive the most adequate interventions recommended for avoiding the worst outcome: maternal death [21]. The role of analgesia in such cases is still not clear, with uncertainties regarding possible increased complications and challenges.

Our data show that only a minority of women included in our study received this intervention at the time of the WHO-MCS survey. All centers included in the WHO-MCS had the capacity to perform a cesarean section; however, capacity to provide analgesia for delivery was not an inclusion criterion. In public health facilities, mainly in low- and middle-income countries, availability of analgesia for delivery may be scarce due to a lack of specialized professionals to provide it.

The low prevalence of women receiving analgesia during childbirth might suggest that best practices during childbirth are not used worldwide; however, this may not be the only explanation. Although labor pain is a physical condition and it has been long recognized as one of the most intense types of pain that can be experienced [22–24], the perception of pain is strongly affected by individual, biological, and psychological characteristics, as also by sociocultural and religious beliefs of women and their communities [25].

Information regarding the prevalence of use of labor analgesia around the world is scarce, with frequencies ranging from 1.4 to 60% in different settings [26, 27]. This may be related to differences regarding choices during childbirth and it is probably determined by nonmedical and biological conditions affecting this phenomenon. Assessing the prevalence of use of analgesia during labor worldwide and the medical or cultural factors associated with use would provide valuable information to health providers and policymakers, to better understand the impact of such a procedure and potentially improve satisfaction.

Our study showed that provision of labor analgesia for women with SMM was very low in less resourced regions of the world (Africa, Asia, and Latin America). However, our data could not assess the reasons for that, which are possibly not only related to the availability of resources but also to personal and community beliefs regarding childbirth. Anesthetic complications are always a concern; however, they are a rare cause of maternal deaths. Their importance as a cause of maternal mortality has been decreasing in the last decades [28].

Perinatal outcomes were similar; among SMM with and without analgesia, however, lack of analgesia was associated with the occurrence of fresh stillbirth and perinatal deaths. We cannot ascertain causality with this study design and most likely such results reflect residual confounding, since poorly equipped facilities cannot offer pain relief to all cases and are unable to provide adequate resources to women with SMM; hence pain relief use is low, and perinatal outcomes are worse.

An interesting finding in our study was that women with higher educational levels are more likely to receive analgesia during labor and vaginal birth. There are no doubts that educational levels are intimately associated with economic conditions and ethnic backgrounds, and previous studies and reviews reported that analgesia was mostly provided for women with better socioeconomic conditions [29, 30]. Our study did not assess the ethnicity of participants included in the study since this data was not recorded. However, considering previous reports [31], probably women of ethnic minorities had less access to analgesia. This kind of inequality needs to be addressed by health workers and policymakers and the first step to do so is to recognize the problem.

Higher educational levels protected women of severe maternal outcomes (maternal death or maternal near miss) in the multivariate analysis that we performed. On the other hand, multiple pregnancies, preterm deliveries, low birthweight, and multiparity were associated with worse outcomes. In the same analysis, analgesia was neither a protective nor a risk factor for the occurrence of severe maternal outcomes. Our data support that analgesia may be offered even for women with severe maternal morbidity, since it will not put them at higher risk for severe maternal outcomes such as death or maternal near miss.

Our study has several limitations. One of them is that we obtained data of a cross-sectional study, and we can only infer associations and no causal relationships. Another limitation is that we only assessed women who delivered vaginally but excluded those who were submitted to a cesarean section due to maternal or fetal conditions. We also did not consider the different types of analgesia for this analysis (systemic, epidural, or spinal were summed). And finally, data collection took place over seven years ago, and possible changes might have happened since then. However, the strength of this study is the number of cases, with a multicounty approach, enabling an overview of analgesia among cases with severe maternal morbidity.

## 5. Conclusions

The overall use of analgesia was for vaginal birth was low, including in women with SMM. There was a significant difference in the use of analgesia comparing cases with and without SMM and the procedure was not associated with increased severity (SMO). Analgesia for childbirth was intimately associated with social characteristics. An effort to expand the availability of labor analgesia in different levels of care should be a concern worldwide particularly in the context of growing CS rates.

## Figures and Tables

**Table 1:** Global occurrence of Severe Maternal Morbidity (SMM) in women who delivered vaginally, the prevalence in different geographic regions of the world and prevalence ratio.

World Region	SMM	Non-SMM	PR (CI)
Asia (125,199)	5,314 (4.2)	119,885 (95.8)	1

Africa (59,155)	2,479 (4.2)	56,676 (95.8)	0.99 [0.83 – 1.19]

Latin America (36,597)	1,995 (5.5)	34,602 (94.5)	1.22 [0.90 – 1.65]

World (221,345)	9,788 (4.4)	211,557 (95.6)	-

**Table 2:** Global performance of analgesia for women with Severe Maternal Morbidity, the prevalence in different geographic regions of the world and prevalence ratio.

World Region (n)	SMM with Analgesia	SMM without Analgesia	PR (CI)
Asia (5,314)	343 (6.5)	4,971 (93.5)	**1**

Africa (2,479)	8 (0.3)	2,471 (99.7)	**0.07 (0.02 – 0.24)**

Latin America (1,995)	250 (12.5)	1,745 (87.5)	1.62 (0.91 – 2.90)

World (9,788)	601 (6.1)	9,187 (93.9)	-

^*∗*^ Statistical test: Chi-square with Yates' correction.

**Table 3:** Sociodemographic and obstetric characteristics of women with SMM according to the performance of analgesia during labor and childbirth.

	With Analgesia	Without Analgesia	Prevalence Ratio (95%CI)
*N*	601	9,187	

*Age (years)* ^*1*^			

<20	74 (12.3)	1,085 (11.8)	1

20 – 34	421 (70.0)	6,742 (73.5)	0.92 (0.54 – 1.58)

≥ 35	106 (17.6)	1,343 (14.6)	1.15 (0.43 – 3.02)

*Marital Status* ^*2*^			

Single	50 (8.3)	954 (10.6)	0.78 (0.44 – 1.36)

Married	551 (91.7)	8,044 (89.4)	1

*Education (years)* ^*3*^			

<5	26 (5.1)	1,902 (22.6)	1

5 – 8	83 (16.1)	2,137 (25.4)	**2.77** (1.46 – 5.27)

9 – 11	136 (26.5)	2,007 (23.9)	**4.56** (2.58 – 8.04)

≥12	269 (52.3)	2,357 (28.0)	**7.60** (4.28 – 13.48)

*Previous birth* ^*4*^			

0	343 (57.1)	3840 (41.8)	1

1 – 2	207 (34.4)	3480 (37.9)	**0.68** (0.57 – 0.83)

≥ 3	51 (8.5)	1861 (20.3)	**0.33** (0.18 – 0.60)

*Previous C-section* ^*5*^			

0	549 (91.8)	8,681 (96.5)	1

1	44 (7.4)	296 (3.3)	**2.18** (1.41 – 3.35)

≥2	5 (0.8)	23 (0.3)	**3.0** (1.49 – 6.03)

Missing: 1 – 17; 2 – 189; 3 – 871; 4 – 6; 5 – 190.

**Table 4:** Prevalence of maternal near miss criteria (organ dysfunction) and maternal death in women with SMM according to the performance of analgesia during labor and childbirth.

Maternal outcome	With Analgesia	Without Analgesia	Prevalence Ratio^*∗*^ (95%CI)
	601 (%)	9187 (%)	

*Maternal near miss criteria according to organ system dysfunction*			

Cardiovascular^1^	16 (2.7)	269 (2.9)	0.93 (0.41 – 2.12)

Respiratory^2^	7 (1.2)	155 (1.7)	0.70 (0.30 – 1.67)

Renal^3^	1 (0.2)	53 (0.6)	0.29 (0.04 – 2.39)

Coagulation^4^	**21 (3.6)**	**152 (1.7)**	**2.15 (1.24 – 3.72)**

Hepatic^5^	2 (0.3)	57 (0.6)	0.55 (0.11 – 2.62)

Neurologic^6^	2 (0.3)	75 (0.8)	0.42 (0.10 – 1.78)

Uterine^7^	**12 (2.0)**	**33 (0.4)**	**5.66 (2.72 – 11.78)**

*Maternal Death*	2 (0.3)	104 (1.1)	0.29 (0.07 – 1.25)

*∗*: reference for all these comparisons was not receiving analgesia.

Missing: 1 – 58; 2 – 55; 3 – 52; 4 – 51; 5 – 50; 6 – 48; 7 – 48.

**Table 5:** Potentially life-threatening conditions (PTLC) associated with analgesia during childbirth in women with severe maternal morbidity and vaginal delivery.

PLTC	With Analgesia	Without Analgesia	Prevalence Ratio (95%CI)
	601 (%)	9187 (%)	

*Hemorrhage*			

Uterine Rupture	6 (1.0)	42 (0.5)	2.18 (0.9 – 5.31)

Postpartum hemorrhage (PPH)	233 (38.8)	2474 (26.9)	1.44 (0.98 – 2.12)

*Infection*			

Puerperal endometritis	8 (1.3)	130 (1.4)	0.94 (0.37 – 2.37)

Pyelonephritis	7 (1.2)	202 (2.2)	0.53 (0.21 – 1.34)

Influenza	3 (0.5)	88 (1.0)	0.52 (0.15 – 1.87)

Other infections	26 (4.3)	465 (5.1)	0.85 (0.35 – 2.11)

*Hypertension*			

Chronic	29 (4.8)	452 (4.9)	0.98 (0.66 – 1.46)

Preeclampsia	145 (24.1)	2385 (26.0)	0.93 (0.74 – 1.71)

Eclampsia	7 (1.2)	339 (3.7)	**0.32 (0.13 – 0.74)**

*Other conditions*			

HIV	4 (0.7)	786 (8.6)	**0.08 (0.03 – 0.23)**

Anemia	90 (15.0)	2030 (22.1)	0.68 (0.39 – 1.19)

Malaria / Dengue	4 (0.7)	160 (1.7)	0.38 (0.07 – 1.96)

Embolic diseases	1 (0.2)	21 (0.2)	0.73 (0.09 – 5.77)

Cancer	1 (0.2)	16 (0.2)	0.96 (0.12 – 7.42)

Heart disease	25 (4.2)	203 (2.2)	**1.88 (1.01 – 3.52)**

Lung disease	17 (2.8)	149 (1.6)	**1.74 (1.02 – 2.99)**

Renal disease	7 (1.2)	92 (1.0)	1.16 (0.47 – 2.90)

Hepatic disease	10 (1.7)	198 (2.2)	0.77 (0.42 – 1.43)

Coincidental disease	12 (2.0)	242 (2.6)	0.76 (0.14 – 4.03)

*Interventions / Management*			

Oxytocin to PPH	197 (32.4)	2136 (23.3)	1.41 (0.93 – 2.13)

Other uterotonics to PPH	32 (5.3)	293 (3.2)	1.67 (0.79 – 3.55)

Magnesium Sulphate	49 (8.2)	1368 (14.9)	0.55 (0.36 – 0.84)

Other anticonvulsant	21 (3.5)	345 (3.8)	0.93 (0.46 – 1.90)

Antibiotics	84 (14.0)	1609 (17.5)	0.80 (0.49 – 1.30)

Blood products	78 (12.6)	1471 (16.0)	0.79 (0.55 – 1.14)

Laparotomy	10 (1.7)	56 (0.8)	2.73 (1.33 – 5.60)

Admission to ICU	31 (5.2)	392 (4.3)	1.21 (0.58 – 2.51)

**Table 6:** Perinatal outcomes in women with severe maternal morbidity and vaginal delivery, according to the performance of analgesia.

Perinatal Outcomes	With Analgesia	Without Analgesia	Prevalence Ratio (CI)
	601 (%)	9187 (%)	

*5-min Apgar Score ≤7*	51 (8.6)	1023 (11.9)	0.72 (0.42 – 1.23)

*Small for gestational age*	**115 (18.9)**	**2647 (29.4)**	**0.64 (0.49 – 0.85)**

*Fresh stillbirth*	**16 (2.6)**	**670 (7.2)**	**0.37 (0.19 – 0.71)**

*Neonatal complications*	100 (16.8)	1208 (13.9)	1.21 (0.75 – 1.95)

*Admission to neonatal ICU*	92 (15.5)	1314 (15.1)	1.02 (0.68 – 1.55)

*Early Neonatal death*	12 (2.0)	266 (3.1)	0.66 (0.28 – 1.55)

*Any adverse perinatal outcome*	119 (19.5)	2204 (23.8)	0.82 (0.57 – 1.19)

*Neonatal near miss* ^*∗*^	128 (21.5)	1447 (16.7)	1.29 (0.91 – 1.85)

*Perinatal death*	**27 (4.4)**	**925 (9.9)**	**0.45 (0.25 – 0.81)**

*Preterm Delivery*	107 (17.6)	1,767 (19.3)	0.91 (0.99 – 1.01)

*Multiple pregnancy*	20 (3.3)	422 (4.5)	0.73 (0.43 – 1.24)

*Fetal presentation*			

Cephalic	585 (96.2)	9,015 (96.6)	1

Breech	23 (3.7)	320 (3.4)	1 (0.98 – 1.01)

*Low birth weight (<2500g)*	115 (18.9)	2,145 (23.2)	0.81 (0.62 – 1.07)

*Congenital malformation*	16 (2.6)	86 (0.9)	**2.86 (1.57 – 5.24)**

Missing

**Table 7:** Multivariate analysis by Poisson's Regression of independent factors associated with SMO (MNM + MD) among women with SMM who delivered vaginally.

Characteristics	Prevalence Ratio	95% Confidence Interval	p-value
Education >8 years	0.43	0.32 – 0.58	<0.001

Multiple pregnancy	1.92	1.30 – 2.83	<0.002

Preterm delivery	1.45	1.16 – 1.81	<0.002

Low birthweight <2500g	1.32	1.03 – 1.69	0.029

Multiparous woman	1.19	1.01 – 1.41	0.039

Other variables tested in this model with no statistical significance: maternal age, marital status, number of previous cesarean-sections, previous maternal comorbidities, performance of analgesia, fetal presentation, and congenital malformation.

## Data Availability

The data that support the findings of this study are available from the World Health Organization but restrictions apply to the availability of these data, which were used under license for the current study, and so are not publicly available. Data are however available from the WHO upon reasonable request and with permission of the director of the WHO-RHR (Reproductive Health Research) unit.
